# Supplementary material for: Deciphering the Molecular Machinery—Influence of sE-Cadherin on Tumorigenic Traits of Prostate Cancer Cells
Source: Biology (Basel). 2021 Oct 7;10(10):1007. doi: 10.3390/biology10101007 (PMC8533516; doi:10.3390/biology10101007)

Supplement S1: Scatterplots

Representative scatterplots from flow cytometry analysis

Integrin expression on PC3 cells related to Figure 5A

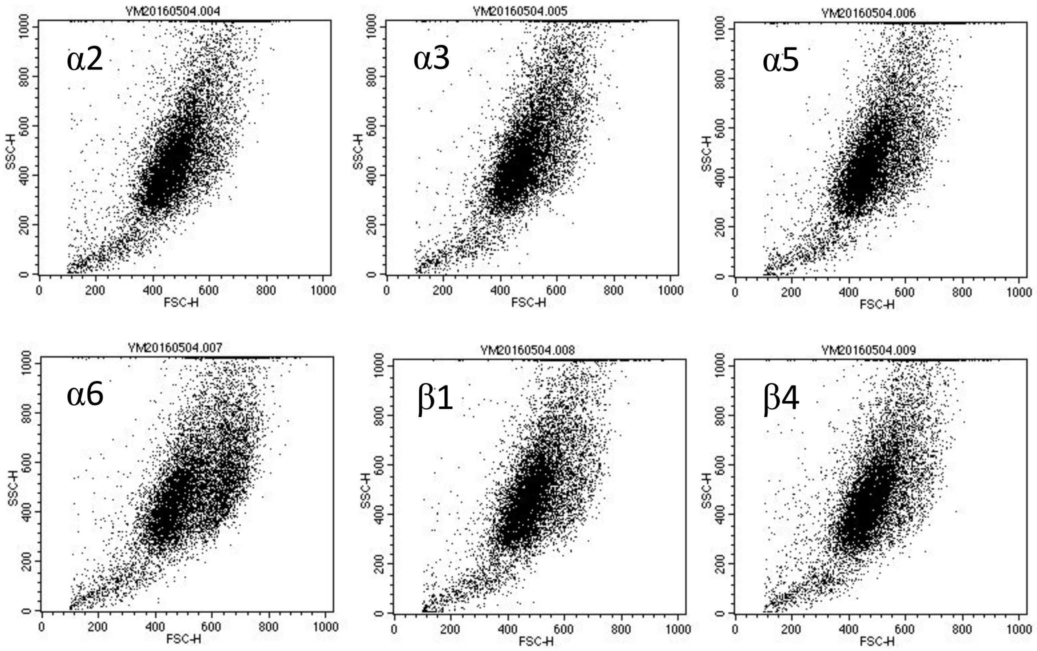

CD44v expression related to Figure 4A

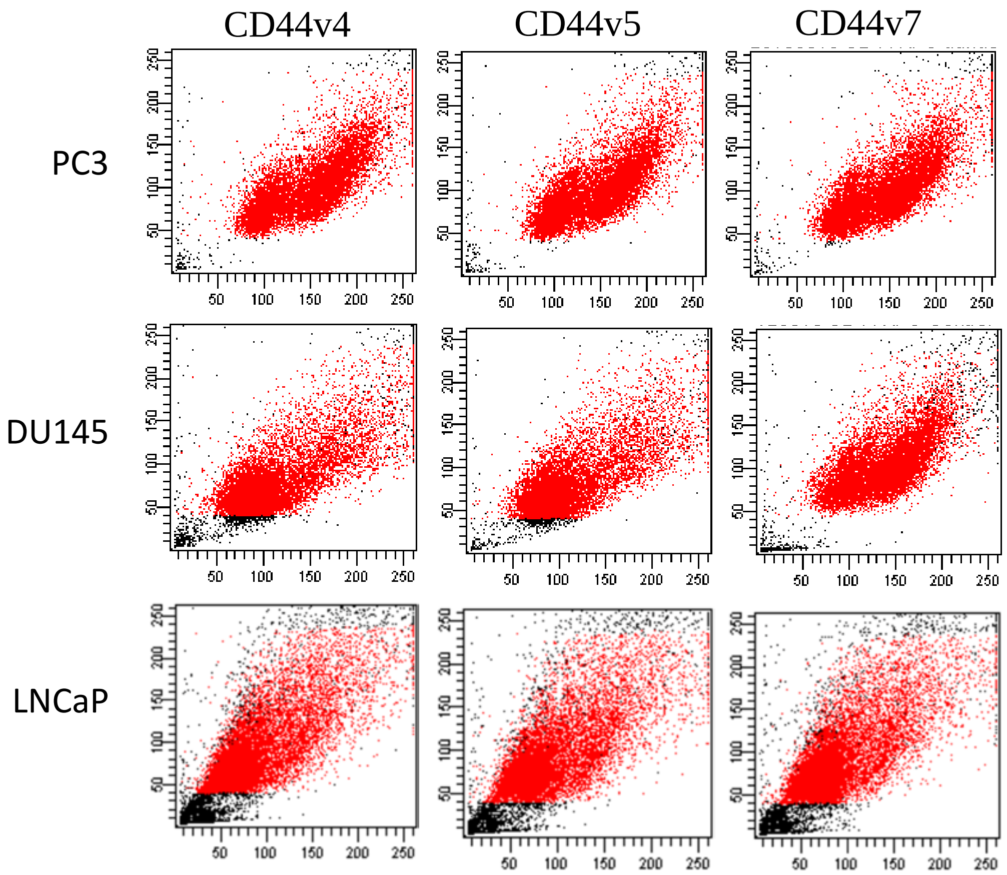

Supplement S2: Protein blots

Figure S5C

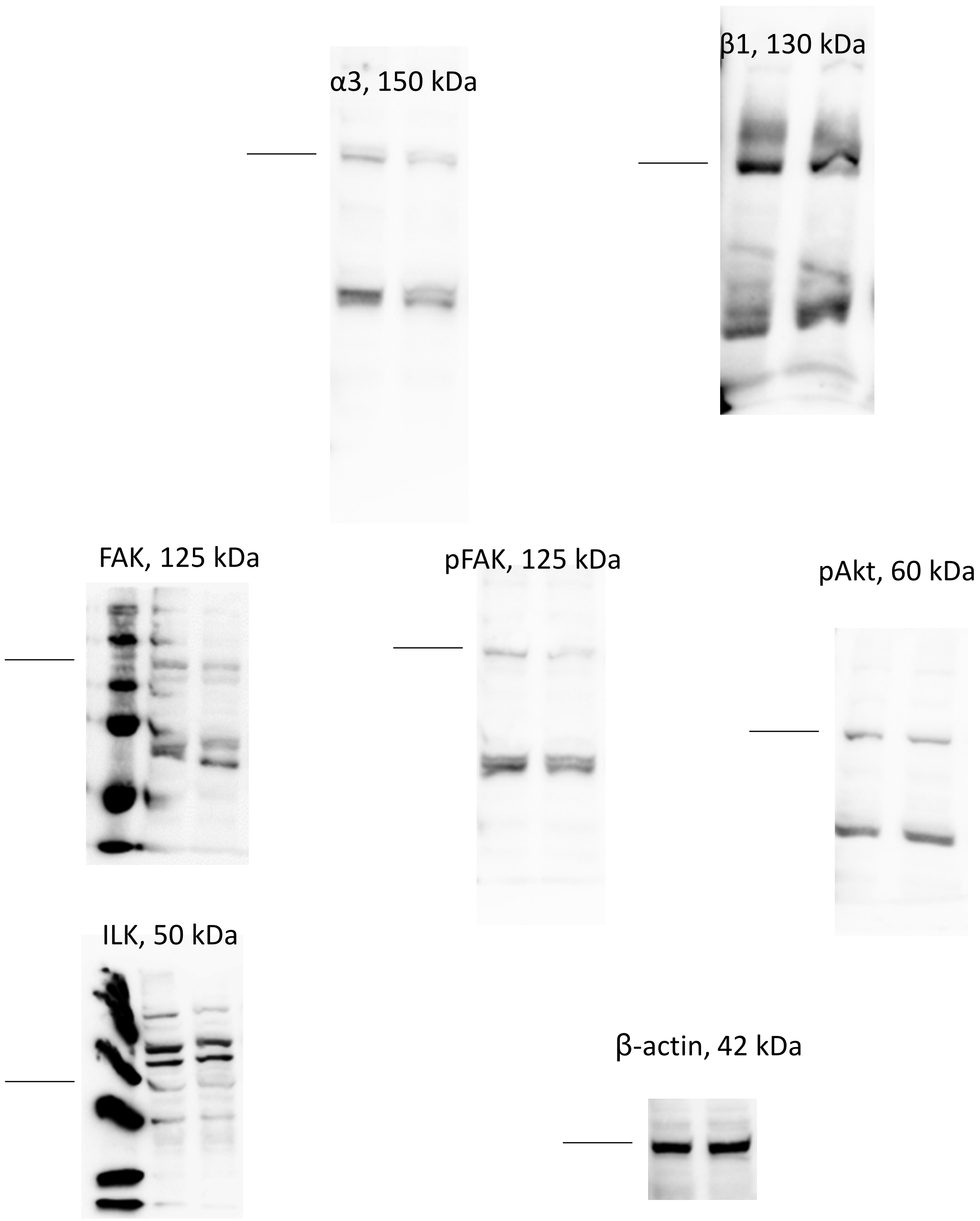

Figure S5F

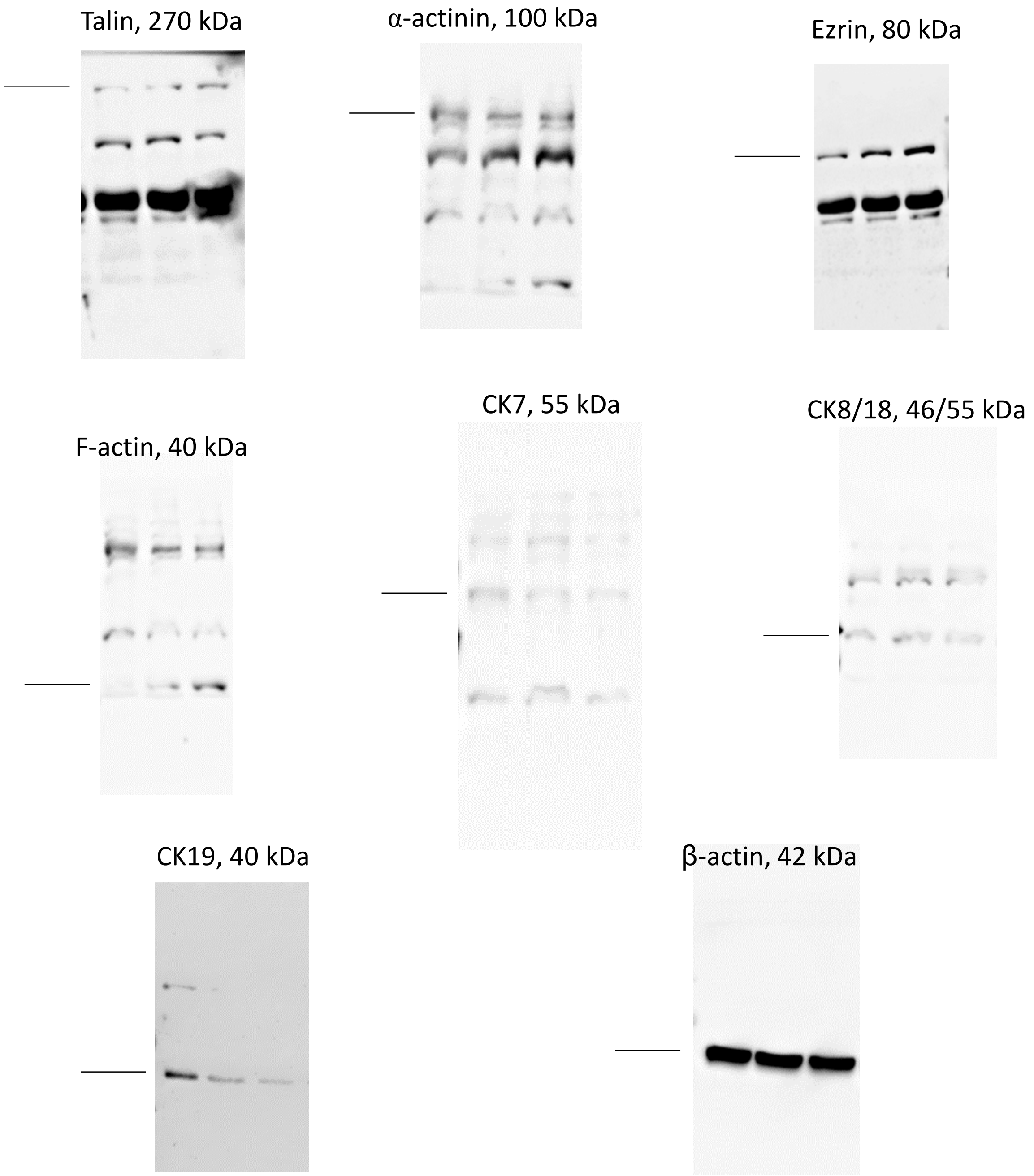

Supplement: Supplementary file 1 [file biology-10-01007-s001.zip › biology-1382076-supplementary-FC.pdf]
